# New Cases of Maleylacetoacetate Isomerase Deficiency with Detection by Newborn Screening and Natural History over 32 Years: Experience from a German Newborn Screening Center

**DOI:** 10.3390/ijns10010017

**Published:** 2024-02-27

**Authors:** Gwendolyn Gramer, Saskia B. Wortmann, Junmin Fang-Hoffmann, Dirk Kohlmüller, Jürgen G. Okun, Holger Prokisch, Thomas Meitinger, Georg F. Hoffmann

**Affiliations:** 1 University Medical Center Hamburg-Eppendorf, Department for Inborn Metabolic Diseases, University Children’s Hospital, Martinistraße 52, 20246 Hamburg, Germany; 2 Center for Pediatric and Adolescent Medicine, Division of Neuropediatrics and Metabolic Medicine, University Hospital Heidelberg, Im Neuenheimer Feld 430, 69120 Heidelberg, Germany; junmin.fang-hoffmann@med.uni-heidelberg.de (J.F.-H.); dirk.kohlmueller@med.uni-heidelberg.de (D.K.); juergenguenther.okun@med.uni-heidelberg.de (J.G.O.); georg.hoffmann@med.uni-heidelberg.de (G.F.H.); 3 Institute of Human Genetics, Klinikum Rechts der Isar, School of Medicine, Technical University Munich, Trogerstrasse 32, 81675 Munich, Germany; s.wortmann@salk.at (S.B.W.); prokisch@helmholtz-muenchen.de (H.P.); thomas.meitinger@mri.tum.de (T.M.); 4 Helmholtz Zentrum München, Institute of Neurogenomics, 85764 Neuherberg, Germany; 5 University Children’s Hospital, Salzburger Landeskliniken (SALK), Paracelsus Medical University (PMU), 5020 Salzburg, Austria

**Keywords:** newborn screening, tyrosinemia type I, succinylacetone, maleylacetoacetate isomerase deficiency, GSTZ1

## Abstract

Newborn screening (NBS) for hepatorenal tyrosinemia type I (HT1) based on a determination of succinylacetone is performed in countries worldwide. Recently, biallelic pathogenic variants in *GSTZ1* underlying maleylacetoacetate isomerase (MAAI) deficiency have been described as a differential diagnosis in individuals with slightly elevated succinylacetone detected by NBS. We report the experience with NBS for HT1 over 53 months in a large German NBS center and the identification and characterization of additional cases with MAAI deficiency, including one individual with a natural history over 32 years. A total of 516,803 children underwent NBS for HT1 at the NBS center in Heidelberg between August 2016 and December 2020. Of 42 children with elevated succinylacetone, HT1 was confirmed in two cases (1 in 258.401). MAAI deficiency was suspected in two cases and genetically confirmed in one who showed traces of succinylacetone in urine. A previously unreported pathogenic *GSTZ1* variant was found in the index in a biallelic state. Segregation analysis revealed monoallelic carriership in the index case‘s mother and homozygosity in his father. The 32-year-old father had no medical concerns up to that point and the laboratory work-up was unremarkable. MAAI has to be considered a rare differential diagnosis in NBS for HT1 in cases with slight elevations of succinylacetone to allow for correct counselling and treatment decisions. Our observation of natural history over 32 years adds evidence for a benign clinical course of MAAI deficiency without specific treatment.

## 1. Introduction

The determination of succinylacetone in dried blood spots (DBS) using tandem mass spectrometry (MS/MS) is a highly effective strategy to identify patients with hepatorenal tyrosinemia type I (HT1, MIM #276700) in newborn screening (NBS) programs [1]. HT1 is caused by a deficiency of fumarylacetoacetate hydrolase (FAH) and leads to severe liver disease, including acute liver failure, cirrhosis, and hepatocellular carcinoma. Detection by NBS allows for early treatment with nitisinone (NTBC) [2]. This treatment, in combination with a diet restricted in tyrosine and phenylalanine, prevents severe liver disease and neurologic complications [3].

NBS for HT1 based on the determination of succinylacetone is performed in several countries worldwide with high sensitivity. Previously, it had been assumed that elevated succinylacetone was pathognomonic for HT1. However, in 2017, Yang and colleagues reported six newborns identified with hypersuccinylacetonemia in the Canadian NBS program who had normal coagulation testing on initial evaluation. These cases did not show disease-causing pathological variants in *FAH* but biallelic pathogenic variants in *GSTZ1*, encoding maleylacetoacetate isomerase (MAAI), the enzyme preceding FAH in tyrosine degradation [4]. These individuals were neither treated with nitisinone nor followed a diet, and a clinical course without relevant medical concerns up to the age of 13 years has been reported [4]. While this suggests a benign clinical course of MAAI deficiency without specific treatment, the long-term consequences of human MAAI deficiency are currently unknown and results from MAAI-deficient mice and one case report imply that under certain conditions, MAAI deficiency may result in clinically relevant hepatic affection [4,5,6,7].

In Germany, NBS for HT1 was started in single NBS centers in the course of pilot studies [8,9], and was included into the national NBS panel in March 2018. Here, we report the experience of succinylacetone-based NBS for HT1 over 53 months at a large German NBS center with the consecutive identification of new cases of MAAI, one of these with a natural history of 32 years.

## 2. Patients and Methods

### Newborn Screening for Tyrosinemia Type I (HT1) Based on Succinylacetone

From August 2016, NBS for HT1 was performed at the Heidelberg NBS center as part of a pilot project for the extension of the German NBS panel (Newborn screening 2020) [9]. Up to December 2020, 379,557 children had participated in this study. From March 2018 on, HT1 was included in the German NBS panel, and the measurement of succinylacetone was performed in all children undergoing NBS. This resulted in NBS data for a total of 516,803 children screened for HT1 between August 2016 and December 2020 at the Heidelberg NBS center.

In Germany, NBS samples are taken between the 36th and 72nd hour of life. NBS from DBS using MS/MS was performed, including electrospray–ionization tandem-MS (Waters Xevo TQD; Waters, Milford, MA, USA) for the determination of amino acids and acylcarnitines, as previously described [10]. At study initiation, the cut-off for succinylacetone in DBS was chosen as percentile (P) 99.5 (1.32 µmol/L). In December 2016, it was increased to P 99.9 (1.59 µmol/L; after method adaption to an underivatized method in March 2020, 2.08 µmol/L).

Exome sequencing and Sanger sequencing were performed as described previously [11,12]. Anthropometric data were evaluated using the following reference percentiles for height [13], body weight [14], and head circumference [15].

## 3. Results

### 3.1. Results of Succinylacetone-Based NBS for HT1 over 53 Months

From August 2016 to December 2016, seven children had false-positive newborn screening results for HT1 (1 in 1485). After increasing the succinylacetone cut-off to P 99.9 in December 2016, another 35 children had abnormal newborn screening results for HT1 (1 in 14,469) over a 4-year period until December 2020. Of these, two children with initial succinylacetone in DBS of 14.15 µmol/L (*N* < 1.59) and 24.52 µmol/L (*N* < 2.08), respectively, were genetically confirmed with HT1. Thirty-one children were classified as false-positive after confirmatory diagnostics, including repeat DBS and an analysis of organic acids in urine. In two children (including the index patient described here), confirmatory diagnostics revealed traces of succinylacetone in urine and further molecular genetic work-up was recommended.

### 3.2. Index Case

The index case is the second child of Afghan parents who are first-degree cousins. The parents themselves and their first child, aged 9 years, were reported to have no medical concerns. The patient was born at 40 weeks gestation after a pregnancy complicated by maternal gestational diabetes treated with insulin. Birth weight was 2570 g (P 1), length 48 cm (P 1), and head circumference 33 cm (P 2). The first days of life were reported to be uneventful. NBS was sampled at 48 h of life and revealed an elevated concentration of succinylacetone in DBS of 2.61 µmol/L, *N* < 1.59. The child was referred to a local hospital for further diagnostic work-up. Liver transaminases and clotting parameters were unremarkable. Samples taken at the age of two weeks showed normalized succinylacetone in DBS of 1.38 µmol/L, *N* < 1.59. An analysis of urinary organic acids showed traces of succinylacetone in urine, quantitatively still below the cut-off of the laboratories’ abnormal range (*N* < 1 mmol/mol creatinine). However, traces of succinylacetone as detected in this individual are usually not found in urine samples of healthy individuals analyzed with this method. 

### 3.3. Molecular Genetic Investigations

**Figure 1:**
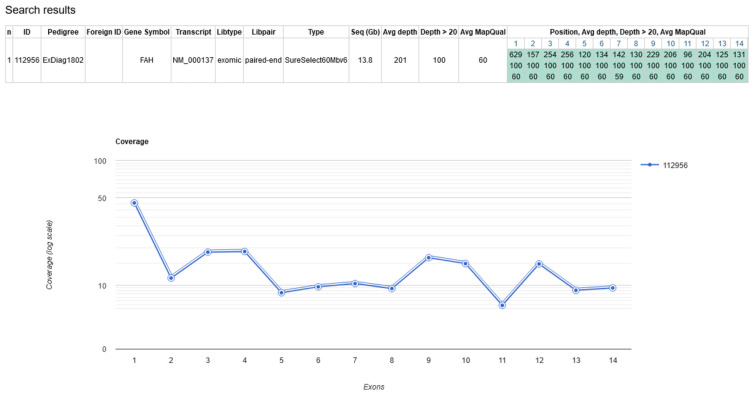
Coverage of *FAH* gene by exome sequencing in index case.

For further diagnostic work-up, a virtual panel from exome sequencing focusing on the genes *FAH* (Tyrosinemia type I, MIM #276700) and *GSTZ1* (Maleylaceoacetate Isomerase Deficiency, MIM #617596) was performed in leucocyte-derived DNA of the index. The entire *FAH* gene was covered well by exome sequencing (average depth 201, all positions had a coverage of >20 reads, Figure 1). The analysis of the *FAH* gene (NM_000137) in the index did not reveal relevant (variants of unknown significance, likely pathogenic or pathogenic) protein-changing variants in coding regions. 

An analysis of *GSTZ1* (NM_145870.2) revealed a homozygous canonical splice site variant c.[136−2A>G]; c.[136−2A>G]; p.[?];[?]. This variant is predicted to lead to a loss of the splice acceptor site in exon 4 and therefore to aberrant splicing and presumably the nonsense-mediated mRNA decay of abnormally spliced protein. The variant was absent from GnomAD and ClinVar databases and rated pathogenic based on the American College of Medical Genetics (ACMG) guidelines [16]. The mother was found to be heterozygous and the father homozygous for this variant. 

### 3.4. Father of Index Patient

The father was without obvious medical complaints at the age of 32 years and adhered to a regular diet including dairy and meat. He had never experienced episodes of liver disease or clinical decompensation despite having experienced several intercurrent infections. The laboratory work-up was within reference range for kidney function, liver transaminases, clotting parameters, and alpha-fetoprotein (AFP; <1.0 IU/mL, *N* < 8). Bilirubin was slightly elevated, with total bilirubin of 1.8 mg/dL (*N* < 1.0) and direct bilirubin 0.4 mg/dL (*N* < 0.3). His amino acid profile in plasma was unremarkable, succinylacetone in DBS was within reference range (0.98 µmol/L, *N* < 1.59), and urinary succinylacetone was undetectable. 

### 3.5. Suspected Additional Case with MAAI Deficiency

The second child from our screening cohort with a possible differential diagnosis of MAAI deficiency also showed slightly elevated succinylacetone in the first and second NBS sample (2.48 µmol/L and 2.47 µmol/L, *N* < 1.59) and traces of succinylacetone in urine at confirmatory testing at age 1 week (2 mmol/mol creatinine, *N* < 1). This child was followed at an external hospital. An analysis of amino acids in plasma at the ages of 2 weeks and 8 weeks showed tyrosine concentrations within the normal range. A follow-up analysis of organic acids repeatedly revealed traces of succinylacetone at the ages of 3 and 6 weeks. Sanger sequencing ruled out disease-causing variants in the *FAH* gene. An analysis of *GSTZ1* was recommended but there is no information available on the results of this analysis.

### 3.6. Treatment and Follow-Up in the Index Patient

No dietary nor pharmacological treatment was initiated in the index patient after the diagnosis of MAAI deficiency. The child received regular infant nutrition without protein restriction. At two months of age, transaminases and clotting parameters were within reference range, total and direct bilirubin were slightly elevated (1.5 mg/dL, *N* < 1.0; 0.4 mg/dL, *N* < 0.3), and AFP was 361.9 IU/mL (N 196–338). The amino acid profile was unremarkable, with tyrosine within the normal range. At follow-up at the age of 7 months, liver and kidney function parameters continued to be unremarkable, and AFP was slightly elevated (41 IU/mL; *N* < 16). While DBS succinylacetone was within the reference range (1.3 µmol/L, *N* < 1.59), the analysis of urinary organic acids repeatedly showed traces of succinylacetone. Due to iron deficiency, the initiation of iron supplementation was recommended. The family was advised to seek medical evaluation, including a determination of liver function and clotting parameters in case of severe intercurrent infections.

At follow-up at the age of 16 months, microcephaly (<P1, −2.68 SDS) with body weight on P15, length on P2, and an otherwise unremarkable physical examination was noted. A developmental evaluation with Denver scales showed age-appropriate development in all domains (social, motor, and speech development). 

A reanalysis of the exome data with respect to variants in known disease genes associated with microcephaly did not show any (likely) pathogenic variants.

Succinylacetone in DBS was 1.01 µmol/L (*N* < 1.59), and the analysis of organic acids in urine continued to show traces of succinylacetone. AFP was normal, with 6.1 IU/mL (*N* < 8).

## 4. Discussion

NBS for HT1 based on the measurement of succinylacetone in neonatal DBS specimens is of significant benefit for affected children as it allows for presymptomatic treatment and has been proven effective to prevent severe liver and neurological disease. The longest running NBS program for HT1 worldwide, in Québec, Canada, has been using succinylacetone as the primary NBS parameter since 1996. This NBS program has recently identified MAAI deficiency as the underlying metabolic cause in several individuals with elevated succinylacetone in NBS. They further reported that affected individuals have remained without relevant medical concerns during a natural history of a maximum of 13 years [4].

Here, we report findings from succinylacetone-based NBS in a cohort of more than 500,000 newborns in Germany. In this cohort, besides two patients with HT1, one individual with confirmed and another with suspected MMAI deficiency were also detected, and one additional case via family screening. In accordance with the report from Canada [4], we chose not to initiate any specific (dietary or pharmacological) treatment in the child with MAAI deficiency in agreement with the family. Follow-up, including neurodevelopmental evaluation, was unremarkable with the exception of microcephaly until the age of 16 months. 

We suggest that confirmatory testing after abnormal NBS for HT1 should include a work-up for MAAI deficiency in cases with a persistent—albeit slight—elevation of succinylacetone without the detection of biallelic pathogenic variants in the *FAH* gene [17]. This is essential for the correct counselling of families and adequate therapeutic decisions, as a biochemical differentiation of both conditions is not reliably possible. Slight elevations of urinary succinylacetone may be found in both mild HT1 and MAAI deficiency, resulting in the recommendation for treatment with nitisinone in the first and—as also supported by our findings—possibly an exclusively observational strategy in MAAI deficiency. Yang and colleagues have suggested that initial treatment decisions concerning the start of nitisinone after abnormal NBS are based on the presence of liver dysfunction based on abnormal coagulations tests. From their experience, all patients with HT1 had abnormal liver function parameters and elevated AFP, in contrast to individuals with MAAI deficiency [4]. In line with this, our patient also showed normal transaminases and clotting parameters and only slightly elevated AFP at the age of 2 months, which normalized within the following months. 

Remarkably, the same genotype as in our index was found in the child’s father, who has remained asymptomatic over 32 years. This is a relevant observation, as to our knowledge it is so far the longest-described natural history for MAAI deficiency. Yang et al. mentioned one 41-year-old individual homozygous for a variant in the *GSTZ1* gene in a database; however, no clinical information was available on this person [4]. The patients in the Canadian cohort were followed for up to 13 years but none of them had a history of serious intercurrent illness (Table 1). They did not receive dietary restrictions, but were discouraged from artificially high protein intake such as the consumption of protein supplements, which would increase the substrate load on MAAI and FAH. In addition, during febrile illnesses it was considered preferable to use non-steroidal anti-inflammatory antipyretics such as ibuprofen over acetaminophen, because of a potential influence of acetaminophen on glutathione levels, with glutathione being a cofactor of MAAI. Yang et al. also recommend avoiding the use of dichloroacetate (DCA) and chloralhydrate, which is degraded in part to DCA, as patients with MAAI deficiency are predicted to be slow metabolizers for DCA [4].

In the individuals with (suspected) MAAI described here, levels of succinylacetone in initial NBS were markedly lower than in HT1 patients from our own NBS cohort and most reported cases of HT1 [18,19,20,21] (Table 2). For the six individuals with MAAI from the Canadian cohort, only plasma levels of succinylacetone after NBS were reported which were also markedly lower than in HT1 [4]. Given the presumed benign nature of MAAI, it could therefore be considered to increase NBS cut-offs for succinylacetone in order to avoid detection of MAAI in NBS. Most published cases of HT1 had initial NBS levels of succinylacetone above 8 µmol/L [18,19,20,21]. However, a comparison of published NBS data has to consider differences in absolute results between derivatized and underivatized methods of MSMS [1,20]. Moreover, single cases of biochemically “mild” HT1 have been published which were diagnosed after clinical manifestation with severe liver disease, but showed only milder elevations of succinylacetone in a retrospective analysis of NBS samples between 4.65 and 5.23 µmol/L [22] or even undetectable succinylacetone in urine with a slight elevation in plasma [23] (compare Table 2). Also, the lower disease range of NBS succinylacetone for patients classified as HT1 in the Region 4S MSMS Collaborative Project comprises cases with succinylacetone as low as 3.8 µmol/L (first percentile of disease range). While it cannot be excluded that cases of MAAI misclassified as HT1 might also be included here, these aspects should be carefully considered by NBS laboratories when discussing an increase in cut-offs for succinylacetone.

The unique position of MAAI deficiency among the disorders associated with phenylalanine catabolism is also evident at the level of genomic variation. Genetic variants underlying life-threatening diseases, unlikely to be transmitted to the next generation, are gradually and selectively eliminated from the population through negative selection. In diploid species, the strength of negative selection at a given locus is predicted to increase with decreasing fitness and increasing dominance of the genetic variants controlling traits [24]. A consensus negative selection score (CoNeS) which integrates intragenic and intergenic sequence variation [24] shows an outlier location for MAAI (+1.87) compared with the other five enzymes involved in the degradation of phenylalanine (Table 3).

The equating of canonical splice variation with a loss of function should consider the isoform spectrum of the gene in question. The adrenal gland produces a GSTZ1 isoform which is not affected by the pathogenic variants and probably also functions as an isomerase. The isoform has a very low expression level, but may potentially show a counter regulation. This aspect should be investigated in the future for an improved understanding of MAAI deficiency.

The results in the father of our index patient, who was never subject to any dietary restrictions and went through several intercurrent infections without notable clinical effect, support the assumed benignity of MAAI deficiency as a mere biochemical condition, as suggested by Yang and colleagues [4]. Increasing cut-offs for succinylacetone prudently could reduce the false-positive rate for HT1 NBS and potentially avoid the detection of MAAI deficiency. Thorough molecular genetic confirmatory work-up is essential to ensure adequate counselling and treatment decisions after positive succinylacetone-based NBS in order to avoid potential over-treatment and the anxiety of parents in this assumedly benign condition. 

## Figures and Tables

**Table 1:** Characteristics of individuals with MAAI deficiency.

Publication	Number of Individuals	Age at Last Follow-Up (Years)	Clinical Information
Yang et al. 2017 [4]	6	1–13 years	No relevant clinical complaints
	1	41 years	None
Gramer et al. 2024 (this publication)	2	16 months; 32 years	No relevant clinical complaints

**Table 2:** Initial succinylacetone levels reported in patients with HT1 compared to individuals with MAAI and controls.

Disorder/Group	Succinylacetone in First NBS DBS (µmol/L)	Succinylacetone in First Plasma Sample after NBS (µmol/L)	Reference
**Tyrosinemia type I** n = 2	14.15/24.52		Gramer et al. 2024 (this publication)
n = 15	Information not provided	16.9–74.4	Yang et al. 2017 [4]
n = 3	10.3–13.7		Cambra Conejero et al. 2020 [18]
n = 4	46–271		Sander et al. 2006 [19]
n = 11	13–81		Turgeon et al. 2008 [21]
n = 1	4.65–5.23		Priestley et al. 2020 [22]
“Mild” Tyrosinemia type I (n = 1)		0.88 (taken age 4 months, *N* < 0.1)	Cassiman et al. 2009 [23]
**MAAI** (confirmed n = 1, suspected n = 1)	2.61/2.48		Gramer et al. 2024 (this publication)
n = 6	Information not provided	0.23–1.28	Yang et al. 2017 [4]
**Controls**	0.21–1.4 (P.1–P.99)		Mc Hugh et al. 2011 [20] (Region 4S MSMS Collaborative Project)
	1.25 (mean)		Turgeon et al. 2008 [21]

Abbreviations: P: Percentile.

**Table 3:** Phenylalanine catabolism, negative selection score, and Mendelian diseases (from [24]).

Substrate	Enzyme	CoNeS	Disease
phenylalanine	PAH	−0.23	PKU
tyrosine	TAT	−0.48	TYRSN2
hydroxyphenylpyruvate	HPD	+0.23	TYRSN3 HWKS
homogentisic acid	HGD	−0.22	AKU
maleylacetoacetate	MAAI	+1.87	MAAID
fumarylacetoacetate	FAH	−0.24	TYRSN1

Abbreviations: CoNeS: consensus negative selection score; PAH: phenylalanine hydroxylase; PKU: phenylketonuria; TAT: tyrosine aminotransferase; TYRSN: tyrosinemia; HPD: 4-hydroxyphenylpyruvate dioxygenase; HWKS: hawkinsinuria; HGD: homogentisate 1,2-dioxygenase; AKU: alkaptonuria.

## Data Availability

The data that support the findings of this study are available from the corresponding author upon reasonable request.

## Abbreviations

DBS	Dried blood spots
HT1	Tyrosinemia type I
MAAI	Maleylacetoacetate isomerase
NBS	Newborn screening
P	Percentile
Succ.	Succinylacetone
